# Optimization of canine sperm cryopreservation by focusing on glycerol concentration and freezing rate

**DOI:** 10.1007/s11259-025-10651-w

**Published:** 2025-01-22

**Authors:** Kazuko Ogata, Ayaka Takeuchi, Shiori Ashibe, Naoko Sugane, Yoshikazu Nagao

**Affiliations:** 1 Faculty of Agriculture, University Farm, Utsunomiya University, Tochigi, 321-4415 Japan; 2 Department of Animal Production Science, United Graduate School of Agricultural Science, Tokyo University of Agriculture and Technology, Tokyo, 183-8509 Japan; 3 Present Address: Institute of Livestock and Grassland Science, National Agriculture and Food Research Organization (NARO), Ibaraki, 305-0901 Japan; 4 East Japan Guide Dog Association, Tochigi, 321-0342 Japan

**Keywords:** Canine sperm, Cryopreservation, Glycerol, Freezing rate

## Abstract

The purpose of this study was to improve the quality of frozen-thawed canine spermatozoa through the optimization of glycerol concentration (GC) and freezing rate in the semen freezing protocol. Ejaculates from nine dogs were diluted with an extender containing 0%, 1.5%, 3%, 6%, or 9% glycerol. The suspensions were loaded into 0.25 ml straws, frozen in nitrogen vapor in a closed box, and immersed in liquid nitrogen (LN_2_). The freezing rate was controlled by setting the distance from the LN_2_ surface to the straws as 1, 4, 7, or 10 cm. Firstly, freezing curves for each GC and freezing rate were analyzed. The analysis showed that the temperature of ice nucleation, freezing point, and immersion were changed with a certain trend depending on the GCs and freezing rates. Secondly, the sperm motility index (MI), viability and mitochondrial (MT) activity were evaluated. At 0 h after thawing, the MI was higher in the 3% and 6% GCs than the 0% GCs (*P* < 0.05). At 24 h, the 3% GC with 1 cm LN_2_ distance (1 cm-3%) and the 7 cm-6% showed higher viability than the other conditions (*P* < 0.05), and the highest MT activity was obtained in the 1 cm-3%, which was higher than the other conditions (*P* < 0.05). The present findings indicate that the rapid freezing rate at 1 cm (average − 31 °C/min) with 3% GC provided the optimal condition in this study; use of this condition should reduce the detrimental damage to dog spermatozoa caused by ice crystal formation during freezing.

**Supplementary Information:**

The online version contains supplementary material available at 10.1007/s11259-025-10651-w.

## Introduction

Improving assisted reproductive technology (ART) for dogs will be useful for the conservation of endangered canine species and for breeding working dogs, e.g., guide dogs for the blind, in which individual genetic traits related to behavioral and personality characteristics are important. Although in vitro maturation of oocytes (Sato et al. 55), in vitro production and cryopreservation of embryos (Abe et al. 1; Nagashima et al. 42), and inducing estrus (Tsuchida et al. 68) have been reported, further developments in ART are still essential for practical use due to the unique reproductive physiology of bitches. Non-surgical artificial insemination using frozen-thawed semen is currently considered a very promising ART technique in dogs (Ogata et al. 44; Suzuki et al. 64); this technique allows genetic improvement from the male side and is also beneficial in terms of conception outcome and animal welfare. However, the decrease in quality of semen after freeze-thawing has become an issue, as it can reduce the likelihood of successful pregnancy in an unpredictable manner (Thomassen et al. 66).

Cryo-injury to the sperm during cryopreservation can cause a decrease in cellular metabolism, alteration to membrane permeability, and the loss of intracellular components (Grötter et al. 19; Hai et al. 21). The quality of frozen-thawed sperm varies among species as the composition of sperm membrane lipids and semen freezability show inter-species differences (Holt 26; Evans et al. 14). Thus, the protocols for other species may not be applicable to dog semen, making it necessary to test a range of modifications to the freezing protocol to identify the optimal conditions for dog semen. Currently, there are considerable differences among canine semen freezing protocols, including extender composition and freezing process (Bencharif and Dordas-Perpinya 6; Suzuki et al. 64) and, as a consequence, post-thaw quality remains variable.

During the freezing process, cellular damage is induced through two distinct mechanisms: ice formation with cell dehydration; and osmotic stress. The extent of the damage due to these factors is related to the cryoprotectant composition and cooling rate (Meryman et al. 40; Mazur 39). Low molecular weight cryoprotectants reduce the damage by intracellular ice formation by replacing intracellular water. Thus, glycerol is able to penetrate into cells and is the major cryoprotectant present in solution (Lovelock 35). In canine sperm cryopreservation, glycerol has been reported to have a greater protective effect for motility than other agents such as methylformamide, dimethylformamide (Futino et al. 18; Hernandez-Aviles et al. 25), dimethylsulfoxide (Lopes et al. 34), and ethylene glycol (Martins-Bessa et al. 37; Kim et al. 31). However, the benefits of glycerol are concentration-dependent: glycerol can cause cytotoxicity and osmotic stress by disrupting phospholipid and protein structures, destabilizing the cell membrane, and altering the cytoplasm viscosity (Amann and Pickett 3; Hammerstedt and Graham 23). A wide range of glycerol concentration from 1 to 16% has been used for canine sperm cryopreservation, and various base extender compositions and addition methods have been investigated (Pena et al. 48; Silva et al. 61, 62, 63; Cardoso Rde et al. 7; Futino et al. 18; Alcantar-Rodriguez and Medrano 2017; Cocchia et al. 10); the optimal concentration of glycerol varies depending on its interaction with the extender composition and freezing rate (Watson 71).

Except for commercial mass production such as for bull semen, sperm freezing generally takes place in small research or breeding institutions and units that do not have programmable freezing equipment for routine use in canines and other mammals (Hori et al. 27; de Paz et al. 12; Darvishnia et al. 11; Ogata et al. 44). In this case, the freezing rate is adjusted by altering the distance between the liquid nitrogen (LN_2_) surface and the straw; however, the freezing rate is also affected by the size of the box and arrangement of the straw mount. Previous studies of canine sperm freezing have tested distances of 2–10 cm (Nothling and Shuttleworth 43; Hori et al. 27; Kim et al. 31; Alcantar-Rodriguez and Medrano 2017), although details of the freezing rate have rarely been analyzed. Therefore, in freezing dog sperm using the box method, the combination of an appropriate glycerol concentration and a freezing rate necessary to provide reproducibility has not been studied in detail and needs improvement.

The objective of this study was to identify optimal conditions for cryopreservation of canine sperm by varying combinations of the freezing rates and glycerol concentrations for reducing detrimental damage associated with cryoinjury.

## Materials and methods

### Animals

Nine, sexually mature male dogs (Labrador Retrievers and crossbred Labrador Retriever x Golden Retriever, 1 to 5 years of age) were used in this study. The males had been used by the East Japan Guide Dog Association as sires in a breeding program to generate guide dogs for the blind. All male dogs have been confirmed their fertility in previous mating.

###  Semen collection and processing

A total of 31 semen samples (1 to 13 ejaculates from each of 9 males) were obtained after digital manipulation in the presence of a bitch in heat as the dummy (Suppl. 1). Considering the adverse effects of the first (pre-sperm) and third (prostatic) fractions (England and Allen 13; Aquino-Cortez et al. 4; Kusum et al. 32), the sperm-rich fraction was isolated and collected with minimizing the contamination of the first and third fractions into the sample. Undiluted ejaculates were transferred into the polypropylene tube immediately after collection and kept in light shielding styrene foam box at approximately 20 °C, for transport to the laboratory (a total period of about 1.5 h). Preliminary tests have confirmed that this transport condition has no negative effect on semen quality (data not shown). In order to use semen of certain criteria, the quality of the fresh semen samples was assessed within 3 h of ejaculation (Suppl. 1). The samples with semen volume 1.7 ± 0.2 ml, sperm concentration 7.4 ± 1.0 × 10^8^ spermatozoa/ml, fresh sperm motility 96.6 ± 0.5% and fresh sperm progressive motility 69.5 ± 3.7% were individually used in the following experiments. The standard Tris–egg yolk–citrate extender supplemented with 5 mM GSH (Ogata et al. 44) was used as the basic extender. For the primary dilution, each ejaculate was divided into aliquots and diluted to obtain a sperm concentration of 2.0 × 10^8^ spermatozoa/ml. The samples were left to equilibrate for 3 h in a refrigerator at 4 °C. An equal volume of the basic extender containing glycerol was then added to obtain a final concentration of 0, 1.5, 3, 6, or 9% glycerol and 1.0 × 10^8^ spermatozoa/ml.

### Freezing and thawing

The sperm samples were loaded into 0.25 ml straws (IMV, L’Aigle, France) and frozen by placing them horizontally in a rack at 1, 4, 7, or 10 cm above the surface of LN_2_ (approximately 4.8 kg) in a closed styrene foam box (22 cm x 26 cm x 23 cm) for 15 min; the straws were frozen in LN_2_ vapor and then plunged into the LN_2_ (Okano et al. 45). The straws were stored in LN_2_ for at least one week before evaluation. Thawing was carried out in a water bath at 70 °C for 5 s (Nothling and Shuttleworth 43), and the thawed sperm samples were placed into sealed 1.5 ml polypropylene tubes and kept at room temperature (approximately 24 °C). All subsequent operations were performed at room temperature unless otherwise stated.

### Production of freezing curves

Temperature changes inside the straw during freezing were recorded every 2 s using an automated measurement program and digital thermometer (MC-3000-000, CHINO, Tokyo, Japan) equipped with a type-K φ0.5 thermocouple (SCHS1-0, CHINO); the recorded data were used to generate freezing curves for each experimental group.

### Assessment of semen quality parameters

Sperm motility was estimated under phase-contrast microscopy (Olympus Optical Co., Ltd, Tokyo, Japan) at a magnification of 200× on a warmed slide (38 °C) at 0, 12, and 24 h after thawing. The entire field in the slide was observed and the motility patterns were classified using the WHO grades with some modifications (World Health Organization 72): +++, progressively motile at a high speed; ++, progressively motile at a moderate or low speed; +, motile without progression; -, immotile. The proportions (%) of sperm in each grade were assessed independently by two observers in order to support objectivity and the average value per sample was recorded as the final motility. The effects of the different treatments were compared using the Motility Index (MI). This index is based on the four patterns of sperm motility and their relative proportions in each group. MI was calculated using a previously described formula (Fukui et al. 17) with some modifications: (% +++ sperm) + (% ++ sperm × 0.75) + (% + sperm × 0.5).

Flow cytometric analyses of sperm were performed in a Cell Analyzer EC800 (SONY, Tokyo, Japan) flow cytometer equipped with a 488 nm laser to evaluate sperm viability and mitochondrial (MT) activity. Green fluorescence (SYBR 14, JC-1 monomer) was measured through a 525 nm band-pass filter, and red fluorescence (PI, JC-1 aggregates) was measured through a 595 nm band-pass filter. To specifically define the settings for the canine sperm population, debris (non-sperm events) were gated out on the basis of forward scatter, side scatter, and electric volume dot plot by drawing a region enclosing the cell population of interest. A total of 9,000 to 10,000 events per sample were analyzed. The data were provided on a logarithmic scale and analyzed using Sony EC800 Flow Cytometry Analyzer software ver. 1.3.5 (SONY).

**Fig. 1:**
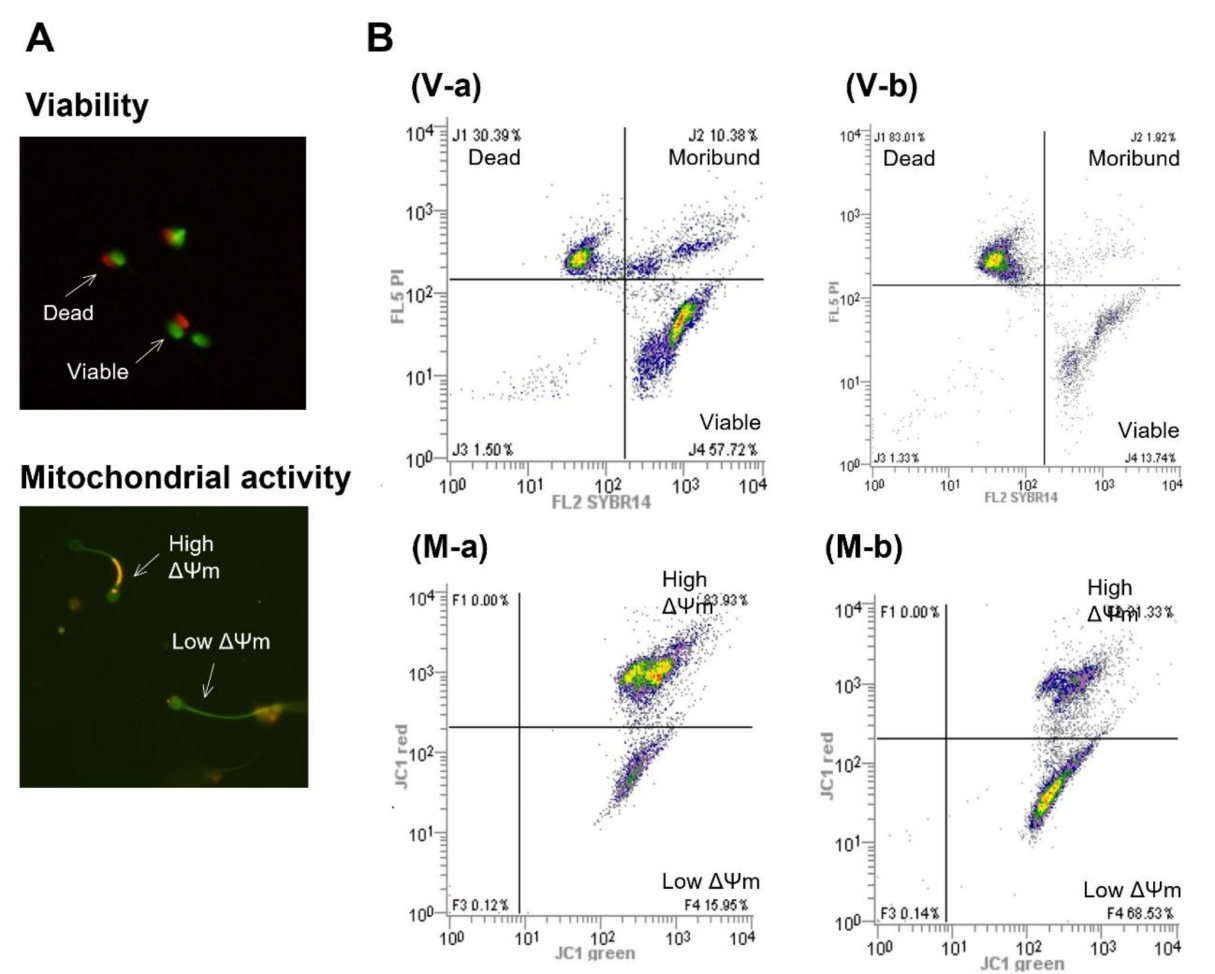
Examples of flow cytometric analyses of dog sperm viability (V) and mitochondrial activity (M). **A**. Fluorescence images of spermatozoa stained with SYBR14/PI (Viability) and JC-1 (Mitochondrial activity). **B**. Representative examples of multicolor cytograms in 0 h (a) and 24 h (b) after thawing. Reference data are derived from the same sample frozen with LN_2_ distance 1 cm and 3% glycerol concentration. (V-a, b) Cytograms of a SYBR14/PI stain. Three populations are identified: dead sperm (red stained), viable sperm (green stained), and membrane-damaged, ‘moribund’, sperm (red/green fluorescence). (M-a, b) Cytograms for the analysis of mitochondrial activity using JC-1. Two populations are identified. Increasing orange fluorescence indicates higher Δψm (mitochondrial membrane potential); green shows the lower Δψm. Unstained debris (low fluorescence) was distributed in bottom left gates and discarded

The viability of the frozen-thawed sperm was assessed using membrane integrity. Samples were evaluated at different incubation times by staining with a Live/Dead Sperm Viability Kit (Molecular Probes, Thermo Fisher Scientific Inc., Eugene, OR, USA) as described in the manufacturer’s guidelines. SYBR 14 (final concentration, 100 nM) and propidium iodide (final concentration, 12 µM), were added to sperm samples, gently mixed, and then incubated for 10 min at 37 °C. Dead spermatozoa (red stain), viable sperm (green stain) and membrane-damaged, ‘moribund’, spermatozoa (red/green fluorescence) were evaluated (Fig. 1A). Aliquots were analyzed by flow cytometer using optimized gating (Fig. 1B).

To estimate MT activity in the spermatozoa, aliquots of each sample were evaluated for mitochondrial membrane potential (MMP) using the lipophilic cationic probe 5,5’,6,6’-tetrachloro-1,1’,3,3’-tetrathylbenzimidazolecarbocyanine (JC-1). The level of JC-1 accumulation reflects the level of mitochondrial membrane activity and enables discrimination of mitochondria with high and low MMPs (Reers et al. 51). JC-1 reversibly changes its fluorescence from the monomeric form (green) when MMP is low to the multimeric form or J-aggregates (red-orange) when MMP is high (Supp2. 1 A). The red/green ratio indicates the MT activity in sperm samples (Volpe et al. 70). The JC-1 Mitochondrial Membrane Potential Assay Kit (Cayman Chemical, Ann Arbor, MC, USA) was used as described in the manufacturer’s instructions. MT activity was evaluated using the flow cytometer and optimized gating (Fig. 1B).

### Experimental design

Experiment 1 investigated variations in the freezing curve and freezing rate at different glycerol concentrations (GCs) and LN_2_ distances. After equilibration, a sensor was inserted into the straw containing semen samples and temperature monitoring was started. Temperature changes during freezing, from the initial equilibrated condition to immersion in LN_2_, were recorded every 2 s. The monitoring was performed 4 times per groups and the averages temperature in the straw at ice nucleation, freezing point, 5 min after freezing point, and immersion were evaluated to obtain the freezing rate. Two aspects of the freezing rate were calculated: from the initial temperature to the ice nucleation point (FR1; (Initial cooling temperature– Ice nucleation temperature) /elapsed time), and serially from the freezing point to 5 min after freezing point (FR2; (Initial freezing temperature– freezing after 5 min) /elapsed time).

In Experiment 2, the effects of different GCs and freezing rates on frozen-thawed canine sperm were investigated to determine the optimal freezing conditions. The time course changes of MI were observed at 0 h, 12 h, and 24 h after thawing. Viability and MT activity were analyzed by flow cytometric analysis at 0 h and 24 h after thawing.

### Statistical analysis

Results are expressed as means ± SEM and statistically analyzed using Statview 5.0 software (Abacus Concepts Inc., Berkeley, CA, USA) and R statistical software (version 4.0.2). Factorial ANOVA with the Tukey-Kramer method was used to compare semen parameters. Differences with values of *P* < 0.05 were considered to be statistically significant.

## Results

### Experiment 1

**Fig. 2:**
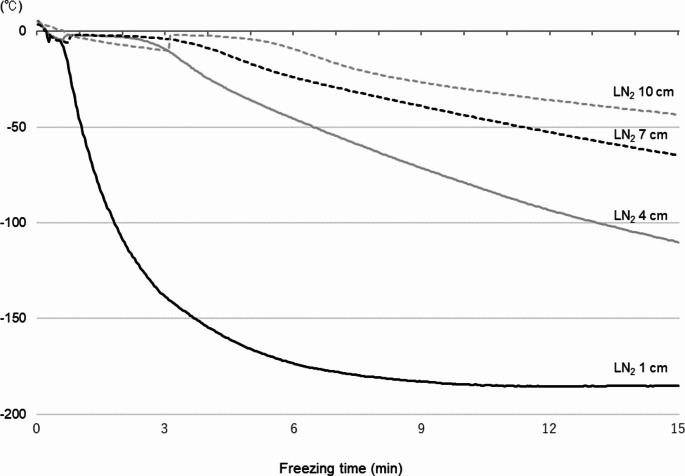
Typical example of freezing curves varying with the distance of LN_2_ surface. Diluted semen samples and thermocouple sensors were loaded into straws, which were frozen above a LN_2_ surface for 15 min before being directly plunged into LN_2_. Temperature changes inside the straws were monitored every 2 s from the start of freezing (cold equilibrium temperature, 4–5 °C) to immersion. The freezing curves of 3% glycerol samples with different LN_2_ distances are shown as a representative example

Representative freezing curves and temperatures for samples containing 3% glycerol are shown in Fig. 2; Table 1, respectively; freezing curves and temperatures for all experimental groups are provided in Suppl. 2. The analyses showed that in all freezing conditions tested, the ice nucleation and freezing point temperatures tended to decrease with increase in GC. The immersion temperatures fell as the LN_2_ distance was decreased regardless of GC. The two components of the freezing rate, namely FR1 and FR2, were obtained separately as shown in Fig. 2; Table 1, and Suppl. 2. The freezing curves at 3% GC shown in Fig. 2 indicate that the smaller the LN_2_ distance, the faster the time of ice nucleation: 16.5 ± 2.3 s at 1 cm, 33.3 ± 7.1 s at 4 cm, 1.1 ± 0.4 min at 7 cm, and 2.5 ± 0.8 min at 10 cm. The latent heat release time, i.e. the time between ice nucleation and the start of the temperature drop recorded as sensible heat, increased with increasing LN_2_ distance: 3.5 ± 0.4 s at 1 cm, 1.6 ± 0.1 min at 4 cm, 2.5 ± 0.4 min at 7 cm, and 3.2 ± 0.4 min at 10 cm. The levels of FR1 and FR2 (Table 1 and Suppl. 2) were mainly determined by the LN_2_ distance, although there were slight differences between the GC groups. The average FR1 for each GC was − 45.6 ± 3.1 °C/min at 1 cm LN_2_ distance, −16.8 ± 1.0 °C/min at 4 cm, −12.7 ± 1.5 °C/min at 7 cm, and − 6.8 ± 1.0 °C/min at 10 cm. The average FR2 for each GC was − 31.4 ± 0.7 °C/min at 1 cm LN_2_ distance, −11.6 ± 0.4 °C/min at 4 cm, −6.2 ± 0.1 °C/min at 7 cm, and − 4.6 ± 0.1 °C/min at 10 cm.

**Table 1:** Average freezing temperatures and freezing rates in samples frozen with 3% glycerol supplementation

LN_2_ distance (cm)	Temperature (°C )			Freezing rate (°C /min)	
	Ice nucleation	Freezing point	Immersion	FR1	FR2
1	−7.2 ± 0.7	−2.8 ± 0.1	−185.5 ± 1.0	−47.3 ± 5.9	−33.4 ± 0.1
4	−5.4 ± 0.6	−4.9 ± 0.4	−125.9 ± 6.7	−19.0 ± 3.0	−13.1 ± 1.1
7	−6.4 ± 1.2	−4.6 ± 0.4	−63.0 ± 0.7	−13.3 ± 3.3	−6.3 ± 0.0
10	−8.7 ± 1.9	−5.2 ± 0.5	−39.3 ± 1.8	−9.7 ± 4.2	−4.4 ± 0.4

The observed temperatures (mean ± S.E.) at three events in freezing and two freezing curves are shown for each LN_2_ distance group. Samples frozen with 3% glycerol supplementation with different LN_2_ distances are shown as a representative example

FR1: Freezing rate 1 represents the time-course changes in temperature during super-cooling, from start point to ice nucleation

FR2: Freezing rate 2 represents the time-course changes in temperature during freezing, from freezing point to immersion

### Experiment 2

**Fig. 3:**
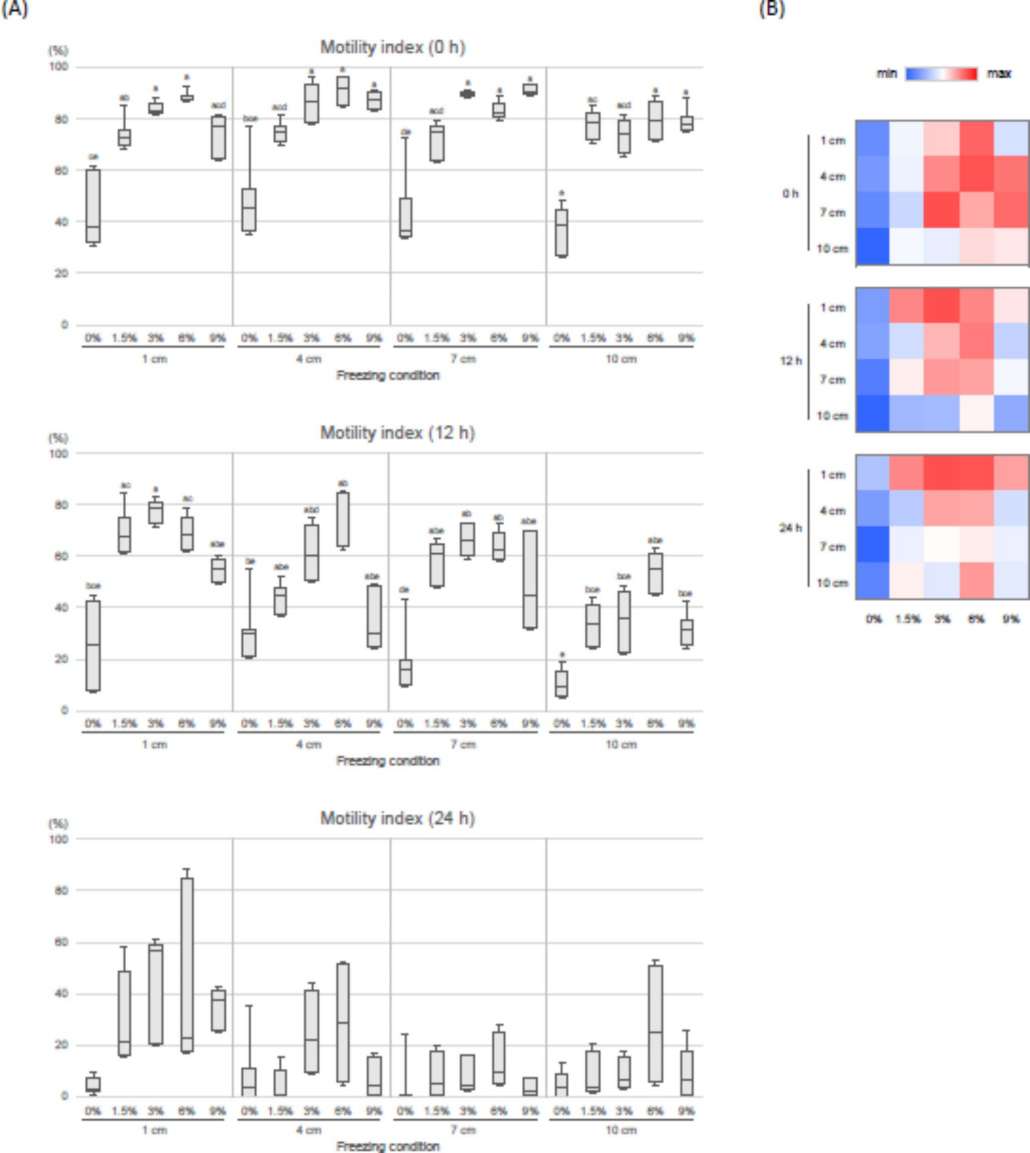
Effects of freezing conditions on the motility index of frozen-thawed dog spermatozoa. Sperm were evaluated at 0, 12, and 24 h after thawing. Three to six replicate experiments were performed. (**A**) Box-and-whisker plots of motility index scores. Mean ± SEM. Different letters (a-e) indicate significant differences (*P* < 0.05). (**B**) Heatmaps of motility index scores. The color scale from blue to red indicates the motility index scores from the lowest to the highest at each time

The effects of different freezing conditions on MI in dog sperm are shown in Fig. 3. At 0 h after thawing, MI was significantly higher in the 3% and 6% GC groups compared to 0% groups (*P* < 0.05) at all LN_2_ distances. At 12 h after thawing, the MI of the 0% and 9% glycerol groups tended to be lower than those of the 1.5%, 3%, and 6% groups. At 24 h after thawing, there were no significant differences among freezing condition, but the 1 cm group tended to be higher than the other distance groups. The highest MIs were observed in the 4 cm-6% group (89.7 ± 4.2%) at 0 h and the 1 cm-3% groups at 12 and 24 h (12 h, 77.6 ± 2.6%; 24 h, 43.3 ± 13.9%).

**Fig. 4:**
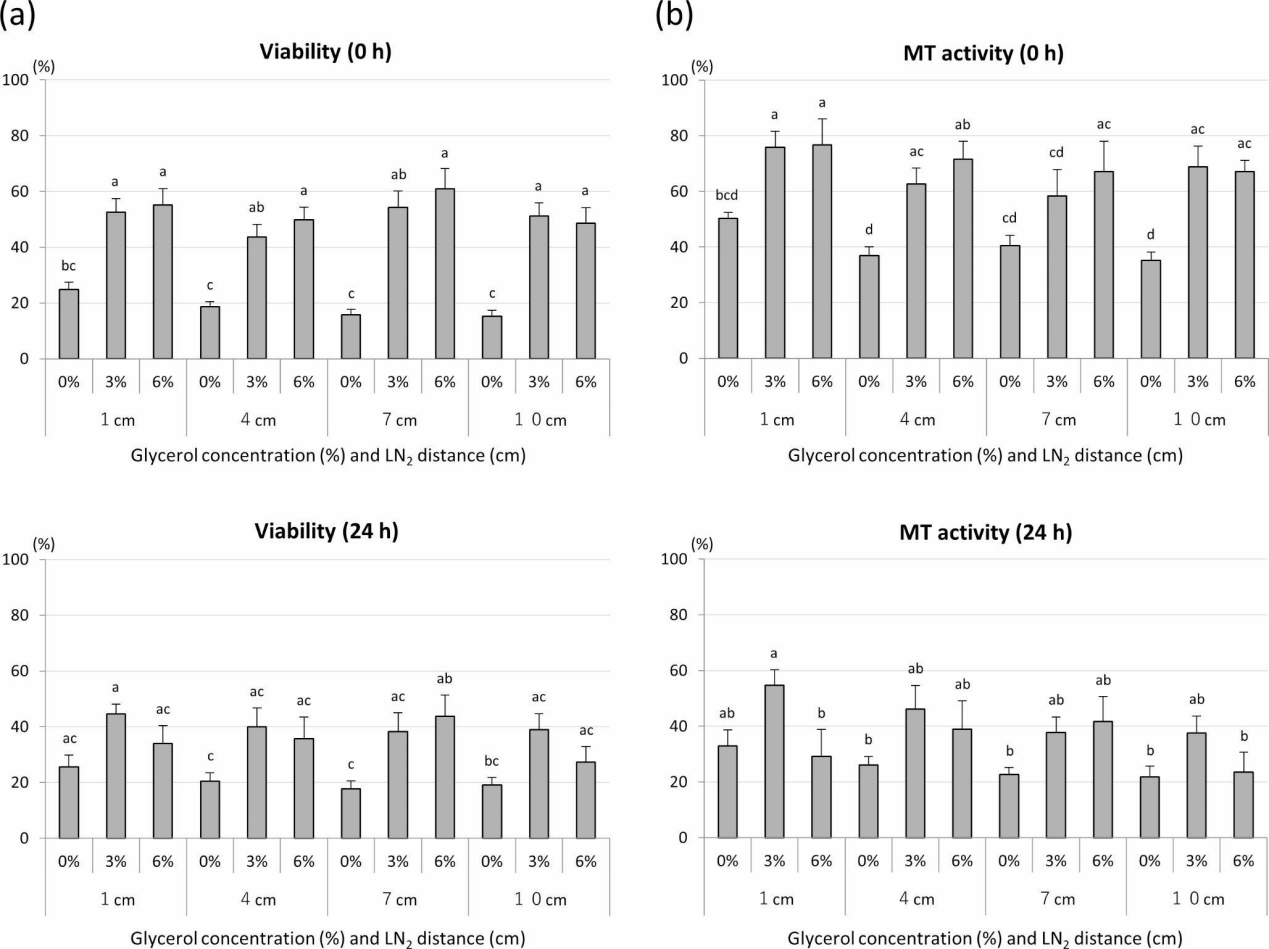
Effects of freezing condition on viability (**a**) and mitochondrial activity (**b**) of frozen-thawed dog spermatozoa. Sperm were evaluated immediately (0 h) and 24 h after thawing. Mean ± SEM. Four to eight replicate experiments were performed. Different letters (a–d) indicate significant differences (*P* < 0.05). MT activity: mitochondrial activity

Viability and MT activity at 0 and 24 h after thawing are shown in Fig. 4. At 0 h, viability and MT activity were higher in 3% and 6% glycerol groups compared to 0% glycerol at 1, 4, and 10 cm distances (*P* < 0.05). At 24 h, the 1 cm-3% and 7 cm-6% groups showed higher viability than the 4 cm-0% and 7 cm-0% groups (*P* < 0.05). For MT activity at 24 h, the 1 cm-3% group showed a higher level (54.8 ± 4.1%) than the 1 cm-6%, 4 cm-0%, 7 cm-0%, 10 cm-0%, and 10 cm-6% groups (*P* < 0.05). There was no difference in MT activity between these latter five groups and other groups.

## Discussion

Ice crystal formation inside and outside the cell during the freezing process causes fatal damage to sperm. In cell cryopreservation, the protective effects of cryoprotectants acting inside and outside the cells and the conditions of cooling and freezing interact with each other (Mazur 39; Varisli et al. 69). We investigated the optimal freezing conditions for canine sperm, focusing on the concentration of glycerol as a cryoprotectant and the distance from the LN_2_ liquid surface to the straws used as containers for the sperm; both factors influence the freezing rate.

In Experiment 1, we monitored temperature changes inside straws during freezing and derived freezing curves for all experimental groups. During the freezing process, as the temperature decreases, supercooling occurs in the diluted semen; subsequently, ice seeding results in the formation of ice crystal nuclei. A transient temperature rise and heat release were observed on the freezing curve due to the generation of latent heat associated with the phase transition. After reaching freezing point, a temperature drop corresponding to sensible heat is initiated, and ice crystal growth is proceeded (Mazur 39; Amann and Pickett 3; Chaveiro et al. 8). Thus, there are two stages in temperature drop on the freezing curve reflecting completely different biophysical events: the pre-ice nucleation (FR1) stage, a liquid phase, and the post-crystallization (FR2) stage, in which ice growth proceeds, and it is reasonable to consider them separately.

Analysis of the freezing curves obtained here showed that ice nucleation and freezing point temperatures appeared to decrease with increasing GC at all LN_2_ distances. This may be a biophysical phenomenon, in which ice seeding and ice crystal growth are influenced by solution composition (Hagiwara et al. 20; Oldenhof et al. 46; Sieme et al. 60), i.e., the freezing point depression due to the presence of glycerol. However, the freezing curves also showed that immersion temperatures at 15 min after the start of freezing converged to similar temperatures for each LN_2_ distance regardless of GC. In a previous report examining freezing times in canine sperm, immersion temperature remained high at around − 20 to −60 °C after 5 min of freezing at a distance of 5, 7 or 10 cm from the LN_2_; this resulted in decreased motility after thawing (Hori et al. 27). In addition, cells can be adversely affected by intracellular ice crystal formation during rapid freezing, as the unfrozen solution remains unstable up to −130 °C (Mazur 39). The immersion temperature of the 10 cm group in this study averaged − 40.3 °C and may have been adversely damaged by the rapid temperature change to −196 °C during immersion.

From the freezing curves obtained in this study, it was not clear whether GC affected the freezing rate or not, as the values varied. In fact, the freezing rate decreased with increasing LN_2_ distance. On the other hand, focusing on no glycerol and 9% glycerol suggested that the presence of glycerol slowed the freezing rate, as observed in the ice nucleation and freezing point temperatures. The FR1 in this study, i.e., the cooling rate during supercooling, was faster than the 2 °C/min and 5 °C/min (Szasz et al. 65) and 3 °C/min (Rota et al. 53) previously applied in dog sperm freezing using programmable freezers. Although optimal sperm freezing protocols vary by species, the physical response of sperm cells to freezing and the mechanisms of damage are thought to be comparable in mammalian species (Morris et al. 30; Sharafi et al. 57). Chaveiro et al. (8) compared FR1 of 1, 4, 20 and 50 °C/min in bovine sperm and reported that the best result was obtained at 4 °C/min, with no significant effect on sperm viability in the 1–50 °C/min range. The FR1 obtained in the present study fell within this range and may not have had a significant negative effect.

The transition from FR1 to FR2 on the freezing curve indicates latent heat release where liquid and ice crystals coexist. In the present study, a longer latent heat release was observed at a slow freezing rate. Thurston et al. (67) reported that the quality of frozen-thawed boar sperm improved when the latent heat release time was shortened. The freezing curves observed in this study suggested that the influence of the latent heat release process may have been low in the 1 cm group.

Our analyses showed an FR2 of −31.4 °C/min at 1 cm, −11.6 °C/min at 4 cm, −6.2 °C/min at 7 cm, and − 4.6 °C/min at 10 cm on average and without considering the effect of GC. In general, the use of a simple freezing method with a styrene foam box provides a slow freezing process when compared to freezing by a programmable freezer (Rota et al. 54). As the freezing rate using the box method is affected by box size and the freezing stand, FR2 freezing conditions reported for canine sperm have varied from − 20 °C/min at 3.5 cm LN_2_ distance (Nothling and Shuttleworth 43) to −12 °C/min (Schafer-Somi et al. 56) or −25 °C/min (Batista et al. 5) at 4 cm. A previous study investigating with a programmable freezer showed that − 0.5 °C/min and − 99 °C/min were too slow and too fast, respectively, while − 12 °C/min and − 28 °C/min showed high motility after thawing (Hay et al. 24). Several reports have shown that freezing in the critical temperature range of −10 to −30 °C at moderately fast freezing rates of −10 to −50 °C/min is effective (Rota et al. 53; Peña and Linde-Forsberg 49). The 1 and 4 cm groups in the present study reproduced these freezing rates.

In Experiment 2, the effect of different freezing conditions on the quality of frozen-thawed sperm was assessed. In this study, the freezing conditions yielding the highest overall quality of sperm were determined by assessing membrane integrity, which can be used to assess damage caused by freezing (Sieme et al. 60), and motility and MT activity, which can be used as indicators of fertilization potential through artificial insemination (Thomassen et al. 66; Meyers et al. 41).

At 0 and 12 h post-thaw, motility was higher in sperm treated with 3% or 6% GC regardless of freezing rate. Motility is a basic sperm quality indicator, and insemination with sufficient numbers of normal, forward-moving sperm is recommended for conception in canine artificial insemination (Mason 38). It has been reported that the cryopreservation process alters the expression of proteins involved in carbohydrate metabolism and glycolytic system, which affect sperm motility, in boar (Chen et al. 9) and ram (Jia et al. 29) sperm. The range of GCs tested the motility in the present study was based on the several reports in dogs, such as 3% (Futino et al. 18), 4% (Cardoso Rde et al. 7), 5% (Alcantar-Rodriguez and Medrano 2017; Cocchia et al. 10), 6% (Silva et al. 61, 63; Cardoso Rde et al. 7), 8% (Pena et al. 48; Cardoso Rde et al. 7), and 12% (Silva et al. 62); however, a reduction in motility was observed in the 9% GC groups at 12 and 24 h. High concentrations of glycerol can cause excessive dehydration, and osmotic stress can disrupt the mechanism and colloidal state of sperm cell membrane (Amann and Pickett 3; Hammerstedt et al. 22). Previously, reduced plasma membrane and acrosomal integrity has been described in 10% and 15% GCs compared to 5% GC in monkey (Si et al. 58), and in 4% and 6% GCs compared to 2% GC in boar (Fiser et al. 15). In addition, the optimal GC may vary depending on the components of extender, cooling rate, and freeze-thaw method (Pena et al. 48). In this study, motility was best maintained with 3% and 6% GCs when using the Tris–egg yolk–citrate-based extender, commonly used in canine semen freezing (Bencharif and Dordas-Perpinya 6). These GCs appeared to be effective in conferring freezing tolerance without causing adverse osmotic effects.

Considering the motility results, we assessed viability and MT activity of sperm frozen in 3% and 6% GCs. Regarding viability at 24 h, the 1 cm-3% GC and 7 cm-6% GC gave better results than the other conditions. With regard to MT activity at 24 h, the 1 cm-3% GC group had the highest (54.8 ± 4.1%) compared to all other groups. The plasma membrane is one of the main sites of lethal damage caused by osmotic changes and reactive oxygen species during cooling, freezing, and subsequent thawing (Ragoonanan et al. 50). A previous study on MT activity in canine spermatozoa showed high MMP even in immobile sperm and suggested that ATP produced by mitochondrial respiration is important for maintaining sperm survival in the female reproductive tract (Volpe et al. 70). Loss of integrity of mitochondrial membranes results in reduced MT activity and release of apoptosis-promoting factors into the cytoplasm, reducing sperm cell longevity (Martin et al. 36).

Differences in frozen semen quality become more apparent during post-thaw incubation (Pace et al. 47; Nothling and Shuttleworth 43). As female dogs ovulate immature oocytes and have a longer optimal fertilization period than other mammalian species, canine frozen-thawed sperm must have long-term viability and motility at the site of fertilization (Rijsselaere et al. 52; Sicherle et al. 59). The maintenance of quality for up to 24 h in the time-course observation in this study suggests that 3% GC may offer sufficient cryotolerance.

Although it is clear from motility analysis that freezing without glycerol is fatal for sperm, there was no significant difference in viability at 0 h in the 1 cm-0% GC group compared to the 4 cm-3% and 7 cm-3% GC groups. In general, the negative effects of rapid freezing include excessive intracellular ice crystal formation due to freezing without sufficient intracellular dehydration (Isachenko et al. 28). However, slow freezing also results in excessive dehydration associated with freezing of the extracellular solution (Foote and Parks 16; Liu et al. 33). Furthermore, ice crystals are destabilized up to −130 °C, and ice crystal growth is active from − 15 to −60 °C, which is considered the danger temperature range for many cells (Mazur 39). Overall, our results suggest that the comparatively rapid freezing in the 1 cm group may have reduced intracellular ice crystal formation and lessened the adverse effects of excessive dehydration. Moreover, this freezing rate allowed the sperm to quickly pass through the detrimental temperature range during the freezing process indicated by FR2 and enabled sperm to be immersed into LN_2_, with a stable intracellular ice crystal.

## Conclusions

In this study, we found that freezing at an average of −31 °C/min, achieved by a combination of 3% GC and 1 cm LN_2_ distance, improved the quality of canine frozen-thawed spermatozoa. It was suggested that under the 1 cm freezing conditions, the spermatozoa quickly passed through the detrimental temperature range of the freezing process and were immersed into LN_2_ in a stable ice crystal state. In addition to this distance condition, low concentrations of glycerol supplementation may have mitigated the adverse effects of the cryoprotectant and of ice formation. Thus, when freezing canine sperm by LN_2_ vapor in a box using glycerol, a mainstream cryoprotectant, use of the freezing conditions described in this study may improve the quality of frozen-thawed sperm.

## Electronic supplementary material

Below is the link to the electronic supplementary material.

Supplementary Material 1

## Data Availability

No datasets were generated or analysed during the current study.
