# Supplementary material for: Optimization of canine sperm cryopreservation by focusing on glycerol concentration and freezing rate
Source: Vet Res Commun. 2025 Jan 22;49(2):86. doi: 10.1007/s11259-025-10651-w (PMC11754312; doi:10.1007/s11259-025-10651-w)
Supplement: Supplementary file 1 — Supplementary Material 1 [file 11259_2025_10651_MOESM1_ESM.docx]

**Suppl. 1. Fresh semen quality.**

|  | n | Semen  volume (ml) | Sperm concentration (x10^8^sperm/ml) | Sperm total motility (%) | Sperm progressive motility (+++%) | Sperm  viability (%) |
| --- | --- | --- | --- | --- | --- | --- |
| Dog 1 | 13 | 1.3 ± 0.2 | 9.3 ± 2 | 97.0 ± 0.8 | 59.0 ± 7 | 96.9 ± 0.6 |
| Dog 2 | 6 | 2.4 ± 0.4 | 9.8 ± 1 | 97.7 ± 0.8 | 83.7 ± 3.9 | 95.0 ± 2.1 |
| Dog 3 | 4 | 1.8 ± 0.5 | 2.8 ± 0.6 | 97.3 ± 1.4 | 76.3 ± 6.6 | 96.3 ± 0.8 |
| Dog 4 | 3 | 1.8 ± 0.3 | 4.5 ± 0.9 | 95.7 ± 0.7 | 71.7 ± 4.5 | 90.2 ± 3.4 |
| Dog 5 | 1 | 1.4 | 5.4 | 95.0 | 50.0 | 97.6 |
| Dog 6 | 1 | 1.7 | 2.7 | 95.0 | 75.0 | ND |
| Dog 7 | 1 | 2.5 | 3.3 | 90.0 | 70.0 | 95.0 |
| Dog 8 | 1 | 1.3 | 5.8 | 93.0 | 85.0 | 95.0 |
| Dog 9 | 1 | 1.9 | 8.2 | 98.0 | 85.0 | 96.3 |
| Total | 31 | - | - | - | - | - |
| Min | - | 0.7 | 1.5 | 90.0 | 15.0 | 84.7 |
| Max | - | 3.8 | 26.5 | 100.0 | 96.0 | 99.2 |
| Mean ± S.E. | - | 1.7 ± 0.2 | 7.4 ± 1.0 | 96.6 ± 0.5 | 69.5 ± 3.7 | 95.6 ± 0.7 |

ND = no data.

**Suppl. 2. Average freezing temperatures and freezing rates at all LN_2_ distances and in all glycerol concentration groups.**

| LN_2_ distance | Glycerol  concentration (%) |  | Temperature (°C) | | |  | Freezing rate (°C/min) | |
| --- | --- | --- | --- | --- | --- | --- | --- | --- |
|  |  |  | Ice nucleation | Freezing point | Immersion |  | FR1 | FR2 |
| 1 cm | 0 |  | -7.1 ± 1.4 | -1.3 ± 0.1 | -185.6 ± 0.7 |  | -46.0 ± 4.3 | -34.1 ± 0.5 |
|  | 1.5 |  | -8.2 ± 0.4 | -2.0 ± 0.1 | -186.6 ± 1.5 |  | -36.8 ± 6.9 | -29.0 ± 1.3 |
|  | 3 |  | -7.2 ± 0.7 | -2.8 ± 0.1 | -185.5 ± 1.0 |  | -47.3 ± 5.9 | -33.4 ± 0.1 |
|  | 6 |  | -11.4 ± 1.2 | -4.3 ± 0.6 | -185.9 ± 0.5 |  | -51.5 ± 5.4 | -33.4 ± 0.3 |
|  | 9 |  | -13.5 ± 0.9 | -6.0 ± 0.3 | -185.7 ± 1.9 |  | -46.6 ± 9.1 | -27.2 ± 1.3 |
|  | Average |  | -9.5 ± 0.7 | -3.3 ± 0.4 | -185.8 ± 0.6 |  | -45.6 ± 3.1 | -31.4 ± 0.7 |
| 4 cm | 0 |  | -3.9 ± 0.7 | -2.4 ± 0.2 | -119.7 ± 5.5 |  | -16.5 ± 1.6 | -12.6 ± 0.7 |
|  | 1.5 |  | -4.7 ± 0.3 | -4.0 ± 0.4 | -105.7 ± 0.5 |  | -17.5 ± 1.8 | -10.8 ± 0.1 |
|  | 3 |  | -5.4 ± 0.6 | -4.9 ± 0.4 | -125.9 ± 6.7 |  | -19.0 ± 3.0 | -13.1 ± 1.1 |
|  | 6 |  | -6.5 ± 0.3 | -6.9 ± 0.2 | -121.5 ± 5.4 |  | -17.5 ± 0.8 | -12.7 ± 0.9 |
|  | 9 |  | -9.6 ± 0.8 | -7.3 ± 0.3 | -103.8 ± 1.0 |  | -14.4 ± 2.4 | -9.6 ± 0.1 |
|  | Average |  | -6.1 ± 0.6 | -5.2 ± 0.5 | -114.1 ± 2.8 |  | -16.8 ± 1.0 | -11.6 ± 0.4 |
| 7 cm | 0 |  | -4.9 ± 0.8 | -2.2 ± 0.2 | -63.2 ± 1.2 |  | -15.1 ± 4.4 | -6.7 ± 0.3 |
|  | 1.5 |  | -4.1 ± 0.1 | -3.8 ± 0.5 | -60.2 ± 0.3 |  | -10.0 ± 1.1 | -6.6 ± 0.1 |
|  | 3 |  | -6.4 ± 1.2 | -4.6 ± 0.4 | -63.0 ± 0.7 |  | -13.3 ± 3.3 | -6.3 ± 0.0 |
|  | 6 |  | -6.9 ± 0.4 | -8.1 ± 0.6 | -62.7 ± 0.8 |  | -16.8 ± 3.8 | -5.7 ± 0.0 |
|  | 9 |  | -7.8 ± 1.0 | -10.1 ± 0.9 | -59.4 ± 0.8 |  | -9.0 ± 1.2 | -5.7 ± 0.2 |
|  | Average |  | -6.1 ± 0.5 | -6.0 ± 0.7 | -61.5 ± 0.5 |  | -12.7 ± 1.5 | -6.2 ± 0.1 |
| 10 cm | 0 |  | -6.2 ± 1.3 | -3.2 ± 0.1 | -42.0 ± 2.6 |  | -9.9 ± 2.2 | -5.2 ± 0.3 |
|  | 1.5 |  | -6.3 ± 1.1 | -3.7 ± 0.3 | -40.8 ± 0.5 |  | -5.4 ± 0.2 | -5.0 ± 0.2 |
|  | 3 |  | -8.7 ± 1.9 | -5.2 ± 0.5 | -39.3 ± 1.8 |  | -9.7 ± 4.2 | -4.4 ± 0.4 |
|  | 6 |  | -7.9 ± 0.3 | -8.0 ± 1.0 | -39.0 ± 0.8 |  | -5.6 ± 0.4 | -4.0 ± 0.1 |
|  | 9 |  | -9.3 ± 0.8 | -7.4 ± 0.4 | -40.3 ± 0.3 |  | -4.6 ± 0.2 | -4.5 ± 0.1 |
|  | Average |  | -7.7 ± 0.6 | -5.5 ± 0.5 | -40.3 ± 0.6 |  | -6.8 ± 1.0 | -4.6 ± 0.1 |

The observed temperatures (mean ± S.E.) at three events in freezing and two freezing curves are shown for each LN_2_ distance and glycerol concentration group. FR1: Freezing rate 1 represents the time-course changes in temperature during super-cooling, from start point to ice nucleation. FR2: Freezing rate 2 represents the time-course changes in temperature during freezing, from freezing point to immersion.
